# Modification of Polyamide Resins by Addition of Polyvinylpyrrolidone

**DOI:** 10.3390/polym17030360

**Published:** 2025-01-28

**Authors:** Yui Ikemoto, Masayuki Yamaguchi

**Affiliations:** 1 Graduate School of Advanced Science and Technology, Japan Advanced Institute of Science and Technology, Nomi 923-1292, Japan; 2 Nippon Shokubai Co., Ltd., Suita 564-0034, Japan

**Keywords:** polyvinylpyrrolidone, polyamide, crystallization rate, viscoelastic properties, polymer blend

## Abstract

The effect of the addition of polyvinylpyrrolidone (PVP) to polyamide resins was studied using polyamide 6 (PA6) and an amide copolymer with a low melting point (PA-L). For the PA6/PVP blends, the crosslinking reaction occurred during rheology measurements in the molten state. The blends did not show a phase-separated structure. Furthermore, the crystallization of PA6 was greatly inhibited by PVP addition. These results suggest that the PVP chains were dissolved in PA6 in the molten state, although the effect of the crosslinking reaction on the structure development is unknown. In the case of the PA-L/PVP blends, melt-mixing and the rheology measurements were performed at low temperature to avoid the crosslinking reaction. It was found that PVP was miscible with PA-L in the molten state when the PVP content was 10 and 15 wt%. The intermolecular interaction between the polyamide resins and PVP was detected from the peak shift of the infrared absorbance. PVP addition enhanced the moisture content in both polyamide resins and decreased the contact angle with water droplets. These results suggested that the surface properties and mechanical properties of polyamide resins, which are affected by the moisture content, are modified by PVP addition.

## 1. Introduction

Conventional polyamide resins, such as polyamide 6 (PA6) and polyamide 66 (PA66), are one of the most successful types of engineering plastics that show excellent mechanical properties and heat resistance [1,2,3,4]. Owing to their strong hydrogen bonds, the crystallization rate of conventional polyamide resins is very fast [5], which sometimes results in poor processability, such as sink marks and warpage in injection molding. In the industry, therefore, the crystallization rate of polyamide resins needs to be decreased in many cases. The addition of nigrosine has been reported to decrease the crystallization rate of PA66 [6,7,8], although it causes discoloration of the product. Another technique to reduce the crystallization rate is the addition of a specific salt [9,10]. It has been reported that the addition of lithium bromide (LiBr) inhibits the crystallization and enhances the glass transition temperature of PA6. It is well known that the lithium cation has a strong interaction with electron-rich parts, such as the oxygen atoms in amide/carbonyl groups [11,12]. As a result, PA6 with 20 wt% LiBr becomes a transparent amorphous material with a high glass transition temperature (ca. 130 °C) [9]. In both nigrosine and salt systems, the hydrogen bonds between polyamide resins and each additive must play an important role in the crystallization kinetics. Polyvinylpyrrolidone (PVP) is also known to decrease the crystallization rate of PA66 [13,14]. The tertiary amide group in the five-membered ring of PVP acts as a proton accepter. Therefore, it shows a strong dipole–dipole interaction with PA66, which reduces the hydrogen bond in PA66. As a result, chain packing to form crystals of PA66 is delayed, leading to slow crystallization.

PVP is an amorphous and non-ionic water-soluble polymer. It is also soluble in various solvents, such as ethanol, propylene glycol, *N*-methylpyrrolidone, and *N*,*N*-dimethylformamide. Because of its good solubility, the electrospinning technique is often used to produce PVP fibers [15,16,17]. Moreover, compared with other water-soluble nonionic polymers, it has good heat resistance under a nitrogen atmosphere. It is also known that PVP undergoes an oxidative reaction at high temperatures under an oxygen atmosphere [18,19].

Although it has been suggested that there is a strong interaction between polyamide resins and PVP, the details of the blend structure, including the miscibility, are still unknown owing to the thermal degradation of polyamide resins [20,21] and oxidative reaction of PVP [18,19]. These reactions may affect miscibility and/or compatibility due to the generation of block or graft copolymers. Moreover, a branch structure produced by the reaction must be responsible for the slow crystallization rate due to a prolonged reptation time. Therefore, the miscibility must be examined without the reactions to understand the structure development and final properties of the blends. Moreover, in the case of PVP addition, the moisture content in the blend product is expected to be affected owing to its strong hydrophilicity, but it has not investigated enough previously [13,14].

In this study, in addition to PA6/PVP blends, which must be very important for industrial applications, we also investigated the structure and properties of blends of PVP and a low crystalline polyamide resin (PA-L) with a low melting point (<120 °C). In the case of PA-L/PVP blends, therefore, we can avoid the effect of oxidation/degradation reactions when the sample is prepared at low temperatures under a nitrogen atmosphere. Furthermore, the effect of PVP addition on the crystallization rate of the polyamide resins was studied. Finally, the moisture content of the blend samples was evaluated by the contact angle with water droplets. Because the mechanical properties of polyamide resins, including the toughness, are known to be greatly affected by the moisture content [1,2,3,4,22], this will be important information for industrial applications.

## 2. Materials and Methods

### 2.1. Materials

Two types of commercially available polyamide resins were used in this study. The first polyamide resin was PA6. The number-average molecular weight of PA6 was 2.2 × 10^4^ Da, which was determined by the end-group determination method. Its melting point *T_m_* was approximately 220 °C. The details of the viscoelastic properties of PA6 have been previously reported [9,10]. The second polyamide resin was an amide copolymer with low crystallinity (PA-L). Its chemical composition, determined by ^13^C-nuclear magnetic resonance spectroscopy, was as follows: PA6 = 55 mol%, PA66 = 13 mol%, and PA610 = 32 mol%. Its number- and weight-average molecular weights, characterized by size exclusion chromatography as the poly(methyl methacrylate) standard, were *M_n_* = 1.1 × 10^4^ Da and *M_w_* = 2.5 × 10^4^ Da, respectively. *T_m_* was located in the broad range from 90 to 120 °C with a small amount of fusion. The details of the rheological and thermal properties have been reported in the literature [23].

Two types of polyvinylpyrrolidone (PVP) with different molecular weights were used: PVP with high molecular weight (PVP-H) and PVP with low molecular weight (PVP-L). The average molecular weights as poly(ethylene oxide) standards were as follows: *M_n_* = 9.9 × 10^3^ Da and *M_w_* = 2.1 × 10^4^ Da for PVP-H and *M_n_* = 3.2 × 10^3^ Da and *M_w_* = 1.1 × 10^4^ Da for PVP-L. The glass transition temperatures (*T_g_*), measured by a differential scanning calorimeter at 10 °C min^−1^, were as follows: 162 °C for PVP-H and 152 °C for PVP-L. The difference in their *T_g_* values is attributed to the difference in their molecular weights [24]. PVP-H was mixed with PA6, while PVP-L with low *T_g_* was mixed with PA-L at a low temperature.

### 2.2. Sample Preparation

After vacuum drying at 80 °C for 12 h, melt-blending was performed using an internal mixer (Xplore MC15HT; Xplore Instrument, Sittard, The Netherlands) at a blade rotation speed of 100 rpm for 1 min. Nitrogen was supplied into the mixer at a flow rate of 200 mL min^−1^ to prevent oxidative degradation. The mixing temperatures were 250 °C for the PA6/PVP-H blends and 200 °C for the PA-L/PVP-L blends. The PVP contents were 0, 10, and 15 wt%. The obtained samples were compressed into flat films using a compression-molding machine at 250 °C for the PA6 blends and at 200 °C for the PA-L blends. Both of the blends were subsequently plunged into an ice-water bath. They were then immediately dried and kept under vacuum conditions prior to the measurements.

### 2.3. Measurements

The morphology of the samples was examined by an atomic force microscope (AFM) (Dimension Icon; Bruker, Karlsruhe, Germany). Prior to the observation, the samples were sliced using an ultramicrotome (EM UC-7; Leica, Wetzlar, Germany) equipped with a diamond knife. A silicon probe (RTESPA-150; Bruker) with a spring constant of 7 N m^−1^ (for the PA6/PVP-H blends) and another silicon probe (RTESPA-300; Bruker) with a spring constant of 40 N m^−1^ (for the PA-L/PVP-L blends) were used for the peak force tapping mode (air) with a scan rate of 0.5 Hz. The scan size was set to 1 μm, and the elastic modulus images were analyzed.

The infrared spectra were collected by attenuated total reflectance Fourier-transform infrared (ATR-IR) spectroscopy (NEXUS 670; Themo Fisher Scientific, Tokyo, Japan) using KRS-5 as the ATR crystal with a resolution of 2 cm^−1^ and 64 scans per sample.

The angular frequency dependence of the oscillatory shear modulus was evaluated at various temperatures using a rotational rheometer (ARES-G2; TA Instruments, New Castle, DE, USA) with a cone-and-plate geometry. The diameter of the cone was 25 mm, and the cone angle was 5.73°. The measurements were performed from high to low frequency.

Thermal analysis was performed using a differential scanning calorimeter (DSC) (DSC 3500; NETZSCH-Gerätebau, Selb/Bayern, Germany) under a nitrogen atmosphere. Each sample (approximately 3 mg) was placed in an aluminum pan and heated from 25 to 250 °C for the PA6/PVP-H blends or 25 to 200 °C for the PA-L/PVP-L blends at a rate of 10 °C min^−1^ and then cooled at 10 °C min^−1^.

The temperature dependence of the dynamic tensile modulus was evaluated from −70 to 225 °C for the PA6/PVP-H blends or from −70 to 125 °C for the PA-L/PVP-L blends using a dynamic mechanical analyzer (RSA-G2; TA Instruments). The measurements were performed at a frequency of 10 Hz and a constant heating rate of 5 °C min^−1^. Rectangular samples cut from the compression-molded films (5 mm wide, 25 mm long, and 0.5 mm thick) were used for the measurements.

The wide-angle X-ray diffraction patterns were collected by an X-ray diffractometer (XRD) (Smart Lab; Rigaku, Akishima, Japan) operating with Cu-Kα radiation.

The hygroscopicity was evaluated using a low-temperature-thermohygrostat (THN064PB; Advantec Toyo, Tokyo, Japan). Square samples cut from the compression-molded films (10 mm wide, 10 mm long, and 0.5 mm thick) were used for the measurements. The samples were placed in a weighing bottle with a diameter of 35 mm. The weight measurements were performed after exposure to the following three conditions: (1) 23 °C and 50% relative humidity (RH), (2) 25 °C and 80% RH, and (3) immersion in water at 23 °C. It was measured three times, and the average value was calculated.

The water contact angles were measured by a contact angle meter (DMo-602; Kyowa Interface Science, Saitama, Japan). The value was recorded 30 s after contact with the surface. The measurements were performed three times, and the average value was calculated.

## 3. Results and Discussion

### 3.1. PA6/PVP-H Blends

**Figure 1:**
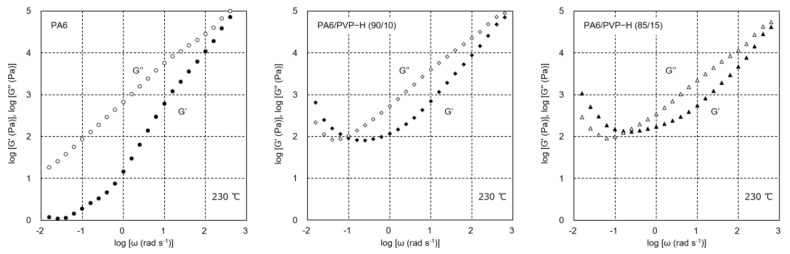
Angular frequency (*w*) dependence of the shear storage modulus *G′* (solid symbols) and loss modulus *G″* (open symbols) of PA6 (**left**), PA6/PVP-H (90/10) (**center**), and PA6/PVP-H (85/15) (**right**) at 230 °C. The measurements were performed from high to low frequency.

The angular frequency dependence of the shear storage modulus *G′* and loss modulus *G”* of PA6, PA6/PVP-H (90/10), and PA6/PVP-H (85/15) in the molten state is shown in Figure 1. The measurements were performed from high to low frequency, and it took approximately 20 min to evaluate the rheological response at the lowest angular frequency, i.e., 0.01 s^−1^.

In the high-frequency region, both moduli of the blends were lower than those of pure PA6. This indicated that PVP-H showed lower moduli at least at 230 °C without crosslinking reaction. In other words, PVP-H acted as a diluent for PA6. PA6 exhibited the typical behavior of the rheological terminal region, i.e., *G″* and *G′* were proportional to *ω*^2^ and *ω*, respectively, at low frequencies [25]. In contrast, the blends showed significantly higher values in the low-frequency region. Considering that the moduli in this region increased with decreasing angular frequency, the crosslinking reaction occurred during the measurements. The reaction was accelerated with a large amount of PVP-H.

**Figure 2:**
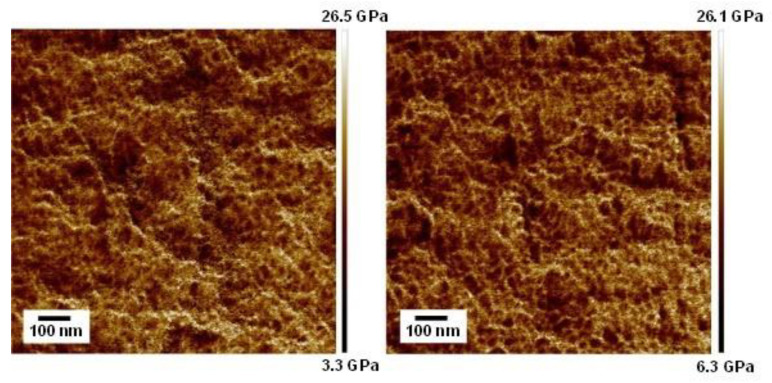
AFM elastic modulus images of the cut surfaces of the compression-molded films of PA6/PVP-H (90/10) (**left**) and PA6/PVP-H (85/15) (**right**).

AFM images of the cut surfaces of the blends are shown in Figure 2. In the figures, the white region represents high modulus. Apparent phase separation was not detected in both blends. The thickness of the white regions, which were needle-shape, was approximately 10 nm. This can be attributed to the crystal lamellae of PA6.

**Figure 3:**
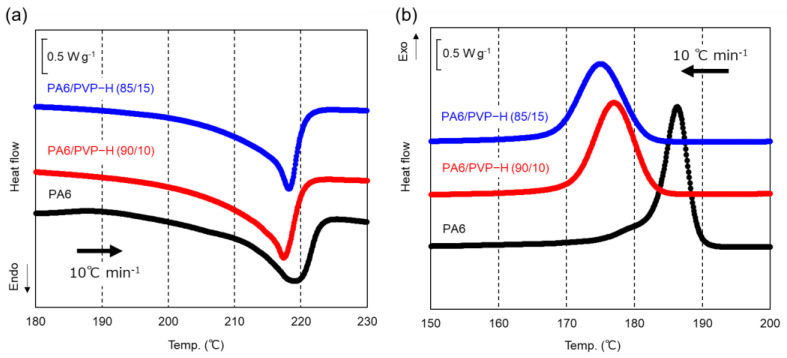
(**a**) DSC heating and (**b**) cooling curves of the compression-molded films of PA6, PA6/PVP-H (90/10), and PA6/PVP-H (85/15) at 10 °C min^−1^.

The DSC heating and cooling curves of the compression-molded films of PA6, PA6/PVP-H (90/10), and PA6/PVP-H (85/15) at a rate of 10 °C min^−1^ are shown in Figure 3. A clear endothermic melting peak was detected at approximately 218 °C for PA6. PVP-H addition barely affected *T_m_* of PA6. The peak temperature of PA6/PVP-H (90/10) was slightly lower than that of PA6/PVP-H (85/15) for unknown reasons. In contrast, PA6 crystallization was greatly inhibited by PVP-H addition. The peak temperatures in the DSC cooling curves were as follows: 186 °C for PA6, 177 °C for PA6/PVP-H (90/10), and 175 °C for PA6/PVP-H (85/15). These results corresponded to those reported using PA66 [13,14,15]. This will be attractive for some processing operations because PVP-H addition can control the crystallization rate of PA6. The delay of the crystallization can be attributed to the mutual dissolution of both polymer chains, because PVP-H with high *T_g_* must increase the viscosity at approximately the crystallization temperature, i.e., decrease the molecular diffusion. Another possibility to inhibit the crystallization is the crosslinking reaction, which provides a long-chain branch structure leading to a prolonged reptation time [26].

**Figure 4:**
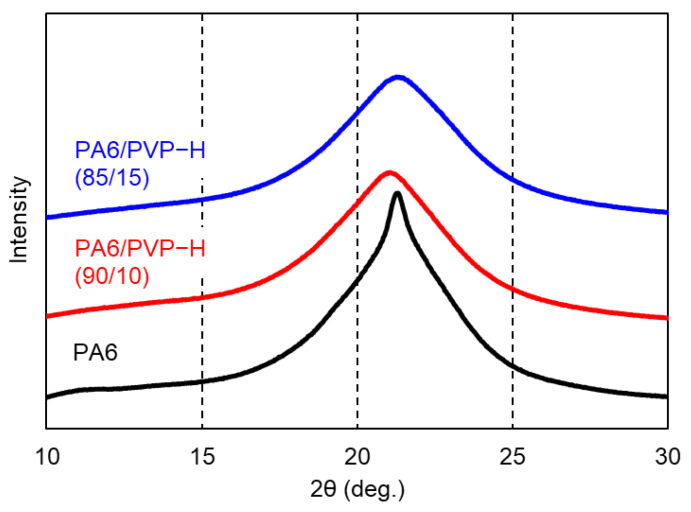
Wide-angle X-ray diffraction patterns of the compression-molded films of PA6, PA6/PVP-H (90/10), and PA6/PVP-H (85/15).

The wide-angle X-ray diffraction patterns of the compression-molded films of PA6, PA6/PVP-H (90/10), and PA6/PVP-H (85/15) are shown in Figure 4. PA6 is a polymorphic material that usually comprises α-monoclinic form and/or γ-pseudo-hexagonal form crystals, which depends on the sample preparation condition [27,28,29]. In these experiments, the γ-form crystals with a strong peak at 2θ = 21.9° of the (200) plane were dominantly formed in the films. This is reasonable because γ-form crystals are generated by rapid cooling [27]. Moreover, a broad peak was detected at a similar 2θ for the blends, presumably owing to less-ordered crystals or a strong contribution of the amorphous region (amorphous halo).

**Figure 5:**
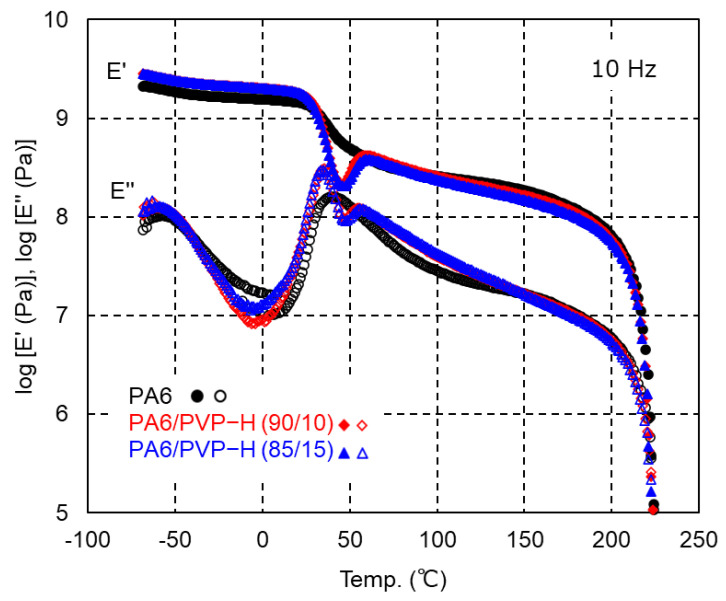
Temperature dependence of the tensile storage modulus *E′* (solid symbols) and loss modulus *E″* (open symbols) at 10 Hz for the compression-molded films of PA6 (black circles), PA6/PVP-H (90/10) (red diamonds), and PA6/PVP-H (85/15) (blue triangles).

The temperature dependence of the tensile storage modulus *E′* and loss modulus *E″* at 10 Hz is shown in Figure 5. Although PA6 showed a monotonical decrease in *E′* with temperature, an increase in the modulus was detected in the blends with PVP-H after the glass-to-rubber transition. This behavior is often detected in a crystalline polymer with a slow crystallization rate [30,31]. When a sample is quenched to a temperature lower than *T_g_* before crystallization, further crystallization is prohibited below *T_g_* due to the restricted molecular motion. During heating, such a sample shows crystallization beyond *T_g_*, which is known as cold crystallization. Sato et al. reported that the addition of LiBr provided the cold crystallization of PA6 [9]. This cold crystallization behavior was also detected in the present samples with PVP-H. Therefore, the results in Figure 5 support the slow crystallization of PA6 with PVP-H addition during the cooling process at the compression molding. Moreover, it should be noted that the *E′* values in the glassy region were enhanced by PVP-H addition. Presumably, PVP-H showed higher *E′* values in the glassy region, although it was impossible to measure the dynamic tensile moduli owing to the brittle nature of the PVP-H film. The peak temperature of the *E″* curve, ascribed to the glass-to-rubber transition, slightly shifted to a lower temperature. The decrease in the crystallinity must be responsible for the *T_g_* shift to a low temperature with a sharp peak of *E″*. Both moduli then sharply decreased at approximately *T_m_*, which was not affected by the addition of PVP-H, similar to the results in the DSC heating curves.

**Figure 6:**
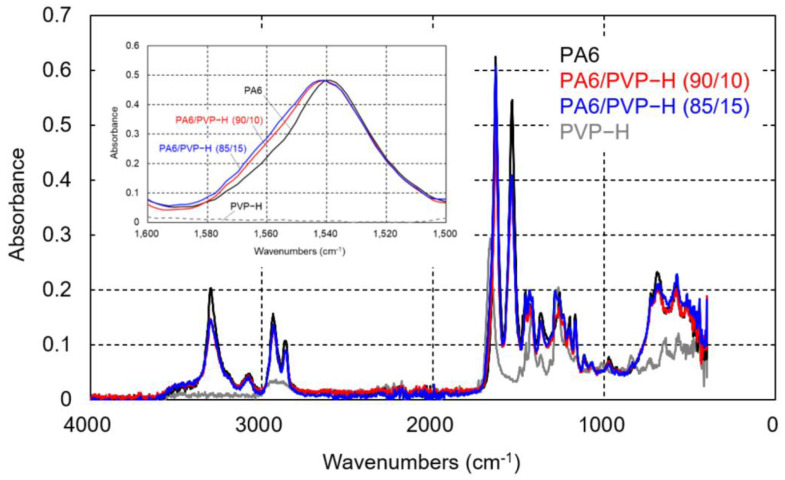
ATR-IR spectra of the compression-molded films of PA6, PVP-H, and their blends.

The intermolecular interaction between PVP-H and PA6 was evaluated by the IR spectra. As shown in Figure 6, the absorbance peak at 1540 cm^−1^, ascribed to the C–N–H in-plane bending mode of PA6 [28,32,33], shifted with the addition of PVP-H, suggesting that intermolecular interaction, i.e., hydrogen bonding, exists between PA6 and PVP-H.

### 3.2. PA-L/PVP-L Blends

The crosslinking reaction cannot be avoided for the PA6 blends because the *T_m_* of PA6 is sufficiently high for the reaction to occur. The long-chain branch structure generated by the crosslinking reaction may play an important role in the crystallization behavior of PA6. Moreover, there is the possibility of producing a block/graft copolymer with PVP-H through the reaction. To discuss the effect of the miscibility on the structure development in the blends, we used PA-L with low *T_m_* to prepare the sample at a low temperature. Furthermore, we used another PVP, i.e., PVP-L, with low *T_g_* because the *T_g_* of PVP-H (ca. 162 °C) is too high to evaluate the rheological properties at low temperatures.

**Figure 7:**
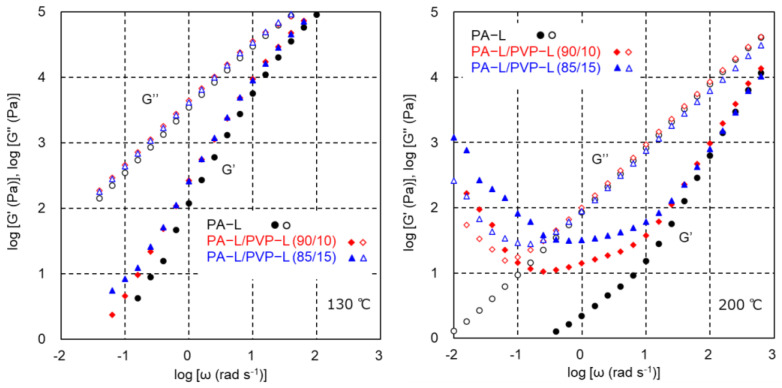
Angular frequency dependence of the shear storage modulus *G′* (solid symbols) and loss modulus *G″* (open symbols) of PA-L (black circles), PA-L/PVP-L (90/10) (red diamonds), and PA-L/PVP-L (85/15) (blue triangles) at 130 °C (**left**) and 200 °C (**right**).

The modulus increase in the low-frequency region was not detected at 130 °C, even for the blend samples, as shown in Figure 7. The *G′* values of the blends were slightly higher than those of pure PA-L in the whole frequency range, which indicated that PVP-L showed high moduli compared with PA-L. There was no clear shoulder in the *G′* curve, suggesting that the system behaved as a simple polymer liquid without any heterogeneous structure. At 200 °C, however, the modulus increase was obvious in the low-frequency region, similar to the PA6 blends at 230 °C.

**Figure 8:**
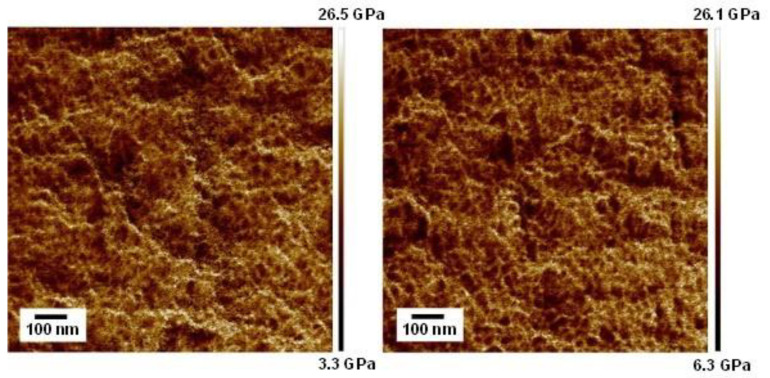
AFM elastic modulus images of the cut surfaces of the compression-molded films of PA-L/PVP-L (90/10) (**left**) and PA-L/PVP-L (85/15) (**right**).

AFM images of the PA-L blends are shown in Figure 8. The phase-separated structure was not detected. This is a reasonable result because there is not a large difference between the solubility parameters of PA6 and PVP [34,35,36,37].

**Figure 9:**
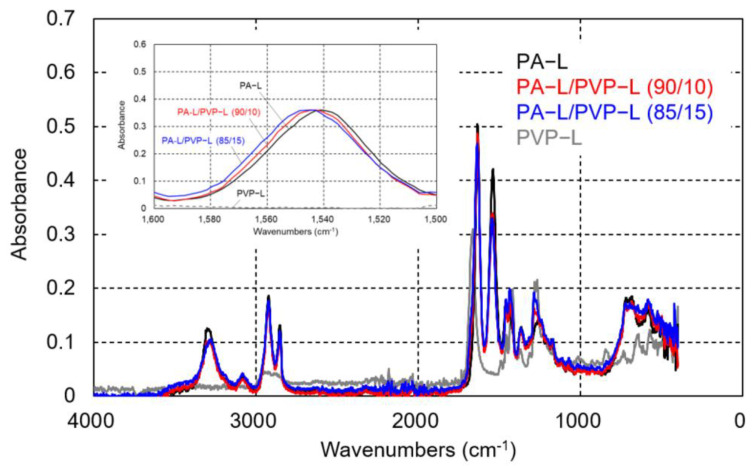
ATR-IR spectra of the compression-molded films of PA-L, PVP-L, and their blends.

The ATR-IR spectra of the compression-molded films of PA-L and the PA-L/PVP-L blends are shown in Figure 9. Similar to the PA6 blends, a peak shift was clearly detected with the addition of PVP-L. Therefore, there must be intermolecular interactions between both molecules.

**Figure 10:**
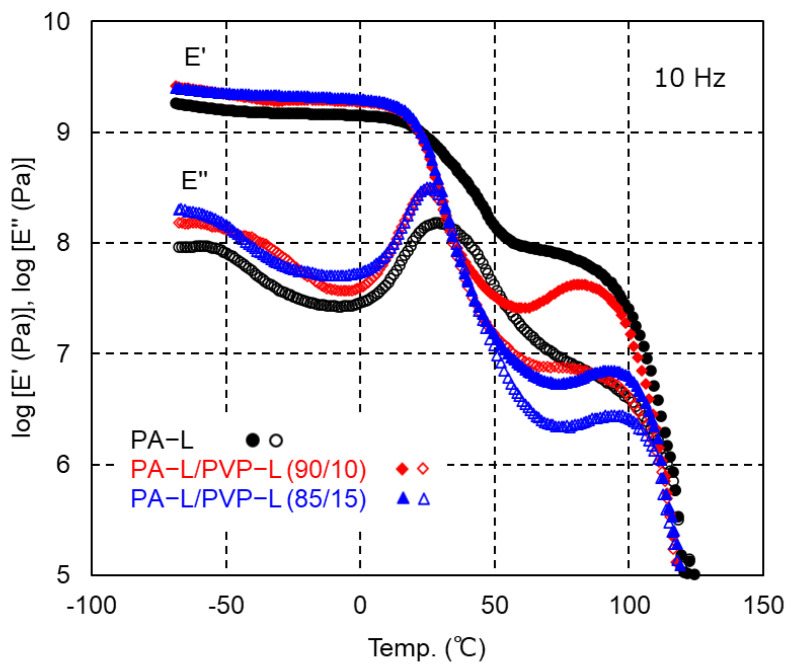
Temperature dependence of the tensile storage modulus *E′* (solid symbols) and loss modulus *E″* (open symbols) at 10 Hz for the compression-molded films of PA-L (black circles), PA-L/PVP-L (90/10) (red diamonds), and PA-L/PVP-L (85/15) (blue triangles).

The dynamic mechanical properties of PA-L and the PA-L/PVP-L blends are shown in Figure 10. Compared with PA6, PA-L showed lower values of *E′* above the glass-to-rubber transition because of its low crystallinity. PVP-L addition further decreased the *E′* values owing to the decrease in the crystallinity. The low crystallinity also affected the shape of the *E*″ peak ascribed to the glass-to-rubber transition, which became sharper with PVP-L addition. The *E′* values increased in the temperature range from 60 °C to 90 °C for the blends. Cold crystallization occurred to some degree during heating. The modulus then greatly decreased at 100 °C owing to melting. Similar to the PA-6/PVP-H blends, *T_m_* of the PA-6/PVP-L blends seemed to be independent of the PVP-L content, although *T_m_* was not clearly detected in the DSC heating curves owing to its low crystallinity.

### 3.3. Water Contents and Surface Properties

Because PVP is highly hydrophilic, it is expected that PVP addition increases the hydrophilicity of polyamide resins. It is well known that absorbed water, which exists in the amorphous region, acts as a plasticizer for polyamide resins. As a result, the mechanical toughness is greatly enhanced [38,39,40]. Furthermore, the water content affects the polarizability anisotropy [9,41]. Therefore, this is an important property.

**Figure 11:**
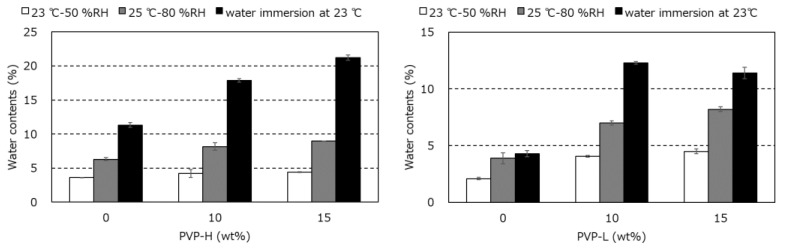
Water contents in the PA6/PVP-H (**left**) and PA-L/PVP-L (**right**) films after storage under various conditions: 23–50% RH (white), 25–80% RH (gray), and immersion in water at 23 °C (black).

The water contents of PA6, PA-L, and their blends after storage under various conditions are shown in Figure 11. The water content greatly increased with PVP addition, and the increase was significant under high-humidity conditions, including immersion in water. It should be noted that the water contents in PA6 and the PA6/PVP blends were higher than those in PA-L and the PA-L/PVP blends, even though the crystallinity of PA6 is higher than that of PA-L. Considering that the water molecules must be in the amorphous region, this was an interesting result. Although the dry process before processing must be important, the plasticization by water is attractive in industrial applications because of the toughness improvement for polyamide resins [38,39,40].

**Figure 12:**
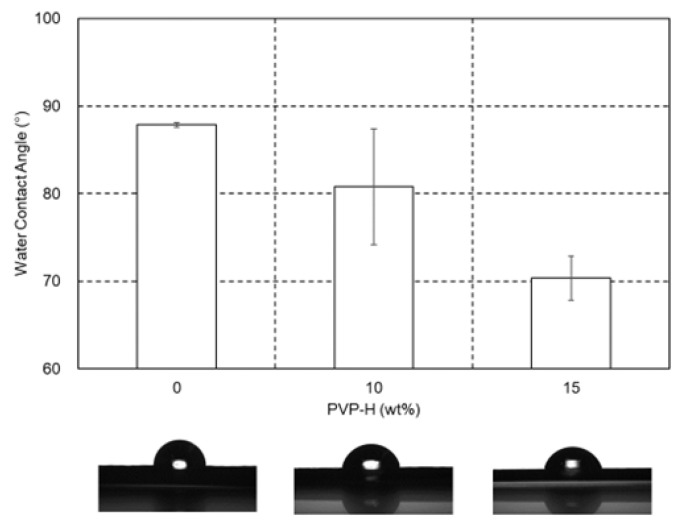
Water contact angles and their pictures of the films of PA6 and the PA6/PVP-H blends at 23 °C and 50% RH.

The water contact angles on the films of PA6 and the PA6/PVP-H blends after storage at 23 °C and 50% RH are shown in Figure 12 with photographs. The water contact angle decreased with increasing PVP-H amount, i.e., the wettability increased. This result demonstrated that the surface properties of PA6 can be controlled by the addition of PVP. In particular, the antistatic property is expected without any additives.

## 4. Conclusions

In this study, the structure and properties of polyamide resins modified by PVP were investigated. PA-L with a low *T_m_* was used in addition to PA6. Because the PA-L/PVP-L blends can be prepared at low temperatures under a nitrogen atmosphere, the effect of the degradation and/or oxidation reactions was ignored. From the viscoelastic properties in the molten state, PVP-L showed miscibility with PA-L when the PVP-L content was 10 and 15 wt%. The interaction between the polymers was indicated by the peak shift in the IR spectrum. PVP-L addition was found to decrease the crystallinity of PA-L, which was also confirmed in the blends of PA6 and PVP-H. In the case of the PA6 blends, the decrease in the crystallization temperature was clearly detected. Because slow crystallization is often required for PA6, especially in injection molding, this will be a good technique for controlling the crystallization rate. PVP addition further affected the moisture content in the polyamide resins. This is important because the mechanical properties of polyamide resins are greatly affected by the water content in the sample. Moreover, the water contact angle decreased with increasing PVP content. These results suggested that PVP addition could become a conventional technique for modifying the surface and mechanical properties of polyamide resins.

## Data Availability

Data are contained within the article.
